# Inflammation-Driven JNK Activation Promotes EMT and Metastasis in Gastric Cancer and Is Attenuated by Huangjin Shuangshen Granules

**DOI:** 10.3390/ph19040636

**Published:** 2026-04-17

**Authors:** Shuo Zhang, Chen Huang, Zhiyuan Song, Jiaheng Lou, Jingcheng Zhang, Sicheng Zhao, Tao Jiang, Guangji Zhang

**Affiliations:** 1 School of Basic Medical Sciences, Zhejiang Chinese Medical University, Hangzhou 310053, China; 2 Zhejiang Key Laboratory of Blood-Stasis-Toxin Syndrome, Zhejiang Chinese Medical University, Hangzhou 310053, China; 3 Traditional Chinese Medicine “Preventing Disease” Wisdom Health Project Research Center of Zhejiang, Hangzhou 310053, China

**Keywords:** HJSS, gastric cancer, metastasis, inflammation, EMT, JNK

## Abstract

**Background:** Gastric cancer (GC) is characterized by aggressive invasion and early peritoneal dissemination, which are strongly driven by chronic inflammation and epithelial–mesenchymal transition (EMT). c-Jun N-terminal kinase (JNK), a stress-responsive serine/threonine kinase within the mitogen-activated protein kinase (MAPK) family, integrates inflammatory cues to promote EMT and metastasis. Huangjin Shuangshen granules (HJSS) is a multi-component traditional Chinese medicine (TCM) formula derived from Simiao Yong’an Decoction and clinically used as an adjuvant therapy for GC. However, whether HJSS restrains inflammation-driven metastasis through modulation of JNK-associated EMT signaling remains unclear. **Methods:** The anti-metastatic efficacy of HJSS was evaluated using integrated in vivo and in vitro models, combined with transcriptomics, network pharmacology and molecular validation. **Results:** HJSS markedly attenuated LPS-induced metastatic behavior and inflammatory activation. Multilevel analyses converged on MAPK8/JNK as a central regulatory node. HJSS reversed EMT progression and inhibited nuclear phosphorylation of JNK without affecting its upstream kinases. Thermal-shift assays and molecular docking supported potential target engagement of HJSS-derived constituents, including possible interactions with JNK-related signaling targets. Pharmacologic reactivation of JNK partially abrogated the inhibitory effects of HJSS, confirming JNK-dependent action. **Conclusions:** HJSS suppresses inflammation-driven GC metastasis primarily by attenuating JNK-associated EMT, potentially through modulation of JNK activation by its bioactive constituents. These findings provide mechanistic insight into HJSS as a low-toxicity anti-metastatic strategy and support further exploration of its active constituents.

## 1. Introduction

Gastric cancer (GC) remains a major global health challenge due to its high prevalence and mortality. According to the latest GLOBOCAN data, there were approximately 968,000 new cases and 660,000 deaths due to GC globally in 2022, ranking fifth in both incidence and mortality among all cancers [1,2]. Owing to its subtle early manifestations, gastric cancer is often diagnosed at an advanced stage. The five-year survival rate for advanced GC remains below 20%, primarily due to its aggressive invasion and high metastatic potential [3,4]. Although advances in targeted and immunotherapies have diversified treatment options in recent years, chemotherapy remains the cornerstone for metastatic GC. Among chemotherapeutic agents, 5-fluorouracil (5-FU) and its derivatives are widely used as part of first-line regimens. 5-FU blocks DNA synthesis by targeting thymidylate synthase, leading to cytotoxic effects [5,6]. However, its clinical utility is often limited by resistance, toxicity, and modest efficacy. Adverse events such as severe myelosuppression, gastrointestinal reactions, and malnutrition are common during treatment [7]. Therefore, strategies that enhance efficacy while minimizing toxicity and delaying resistance are urgently needed to optimize chemotherapy for GC metastasis.

EMT is considered a key event in the metastatic cascade of GC. During EMT, epithelial cells lose cell–cell adhesion and gain mesenchymal traits, enabling them to breach the basement membrane and enter the bloodstream, thereby accelerating tumor dissemination [8,9,10,11]. Inhibiting EMT is thus a promising strategy to prevent GC metastasis. The c-Jun N-terminal kinase/mitogen-activated protein kinase (JNK/MAPK) pathway, a conserved serine/threonine kinase cascade, is activated by stress and inflammatory signals and is crucial for EMT regulation [12,13,14].

Upon activation by factors such as inflammation, stress, and growth factors, this pathway modulates transcription factors such as Snail, Slug, Twist, and ZEB1, driving EMT and enhancing metastatic potential [15,16]. Moreover, chronic inflammation is increasingly recognized as a key driver of GC progression. In particular, *Helicobacter pylori* infection and persistent gastric mucosal inflammation significantly elevate the risk of metastasis [17,18]. Lipopolysaccharide (LPS), a bacterial endotoxin, is frequently used to mimic inflammatory conditions and can activate the JNK/MAPK pathway via the TLR4 receptor, promoting tumor progression [19,20,21]. Thus, targeted inhibition of the JNK/MAPK pathway presents a viable strategy for controlling GC metastasis.

Traditional Chinese Medicine (TCM), with a history of millennia in clinical application, represents a well-established medical system. In recent years, TCM has gained global attention as a complementary and alternative approach, particularly for its roles in modulating EMT, suppressing metastasis, and improving the inflammatory tumor microenvironment [22,23,24]. For instance, components such as *Astragalus membranaceus (Fisch.) Bunge*, *Salvia miltiorrhiza Bunge*, and *Actinidia chinensis Planch* have been shown to regulate cancer-related signaling pathways and exert anti-tumor effects [22,25]. Huangjin Shuangshen Granules (HJSS), a compound TCM formulation, is derived from the classical prescription Simiao Yong’an Decoction recorded in Yanfang Xinbian, with the addition of *Astragalus membranaceus (Fisch.) Bunge*, *Salvia miltiorrhiza Bunge*, and *Actinidia chinensis Planch*. It has shown potential in modulating the inflammatory microenvironment and enhancing immune function. HJSS is a multi-component herbal formula with a chemically diverse composition. Based on the UPLC-QTOF-MS analysis in the present study together with published phytochemical studies on its component herbs, HJSS mainly contains flavonoids, phenolic acids, diterpenoid quinones, and triterpenoid saponins. Representative compounds such as luteolin, quercetin, kaempferol, chlorogenic acid, salvianolic acids, and cryptotanshinone have been reported to possess anti-inflammatory and anti-tumor activities, including modulation of MAPK/JNK-related or other cancer-associated signaling pathways [26]. In the present study, several of these compounds were experimentally detected and further implicated by network pharmacology and docking analyses, supporting the view that the pharmacological effects of HJSS may result from the coordinated actions of multiple bioactive constituents rather than a single compound.

This study investigated the suppressive effects of HJSS on GC metastasis and further elucidated its underlying molecular mechanisms. Our work systematically profiled the chemical components of HJSS using ultra-performance liquid chromatography–quadrupole time-of-flight mass spectrometry (UPLC-QTOF-MS), and several representative constituents were tentatively identified and structurally annotated. Building upon this foundation, we established an LPS-induced GC metastasis model and evaluated the impact of HJSS on EMT in both cellular and animal models. Network pharmacology and transcriptomic analyses were employed to identify potential therapeutic targets and signaling pathways, with particular focus on the JNK/MAPK pathway. Our findings not only reveal the molecular mechanisms of HJSS in GC metastasis but also provide theoretical support for its potential clinical application.

## 2. Results

### 2.1. UPLC-QTOF-MS Analysis Reveals Diverse Bioactive Constituents in HJSS

**Figure 1:**
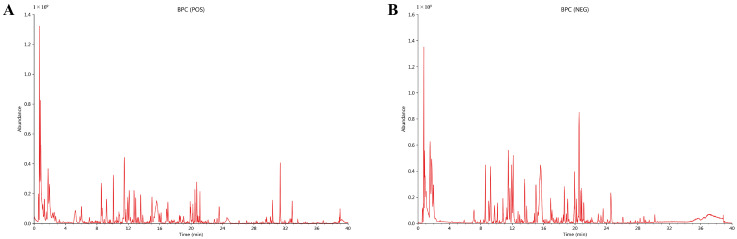
UPLC–QTOF–MS profiling of HJSS. (**A**) Base peak chromatogram (BPC) acquired in positive ion mode (ESI+). (**B**) Base peak chromatogram acquired in negative ion mode (ESI−).

To characterize the chemical composition of HJSS, UPLC-QTOF-MS analysis was performed in both positive and negative ion modes. As shown in Figure 1A,B, the base peak chromatograms (BPCs) exhibited rich and well-resolved peaks across the chromatographic run, indicating the presence of multiple constituents with diverse physicochemical properties. Distinct peak distributions were observed in the positive and negative ion modes, reflecting complementary ionization behaviors of different compound classes within the HJSS formulation.

Further annotation of the LC-MS data identified multiple representative constituents in HJSS, among which salvianolic acid Y, secologanic acid, chlorogenic acid, neochlorogenic acid, cryptochlorogenic acid, isochlorogenic acid C, rosmarinic acid, and cryptotanshinone were among the relatively abundant components detected (Table S1). These findings indicate that HJSS contains multiple chemically distinct classes of compounds, including flavonoids, phenolic acids, diterpenoid quinones, and saponins.

Importantly, several representative compounds involved in subsequent docking analysis, including luteolin, quercetin, kaempferol, cryptotanshinone, and isorhamnetin, were experimentally detected in HJSS (Table S2), thereby linking the chemical profiling results with the downstream network pharmacology and target-interaction analyses. In addition, the detected constituents could be traced to the major component herbs of the formula, further supporting the traceable chemical composition of HJSS. To improve annotation transparency, representative major constituents were further summarized in Table S3, including comparison of theoretical and experimental precursor masses, mass errors, and characteristic fragment ions.

### 2.2. HJSS Suppresses LPS-Driven Peritoneal Metastasis and Alleviates Systemic Injury In Vivo

**Figure 2:**
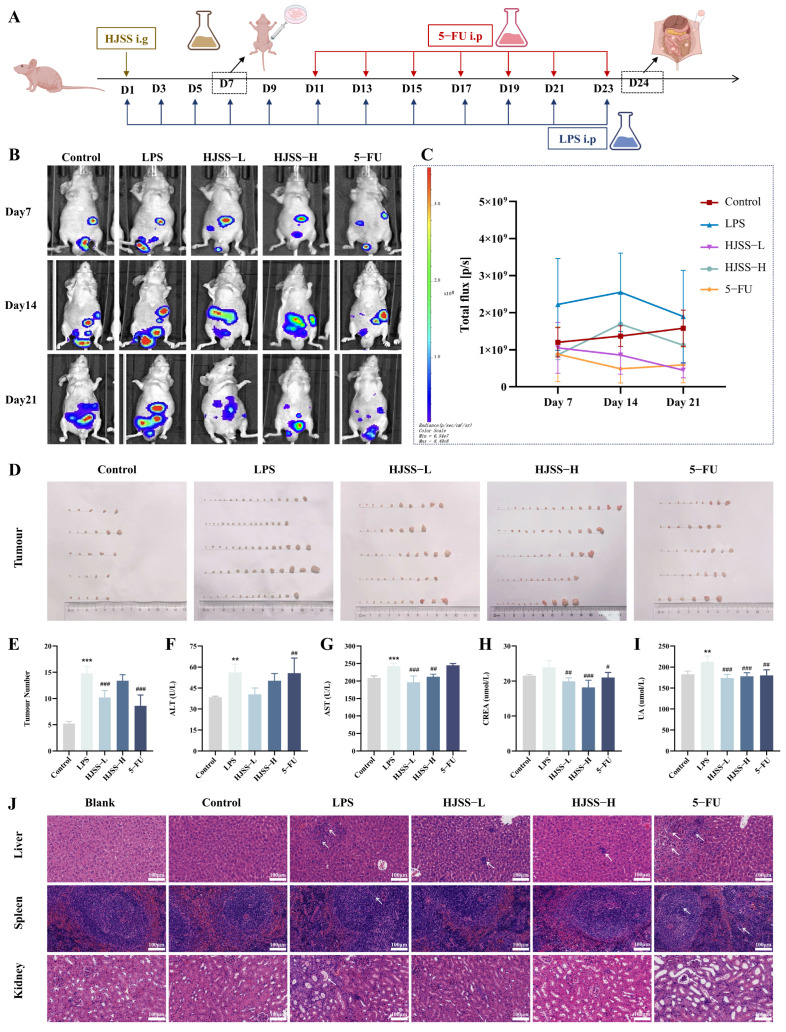
HJSS suppresses intraperitoneal gastric cancer metastasis and improves hepatic/renal function in nude mice. (**A**) Schematic timeline of model establishment and treatments. (**B**) Representative bioluminescence imaging of intraperitoneal tumor burden at days 7, 14, and 21. (**C**) Quantification of total bioluminescence intensity. (**D**) Representative images of intraperitoneal tumor nodules. (**E**) Quantification of intraperitoneal tumor nodules. (**F**–**I**) Serum biochemical indicators reflecting liver and kidney function. (**J**) Representative H&E staining of liver, spleen, and kidney tissues. Data are presented as mean ± SEM, *n* = 5. ** *p* < 0.01, *** *p* < 0.001, vs. Control group, ^#^ *p* < 0.05, ^##^ *p* < 0.01, ^###^ *p* < 0.001, vs. LPS group.

To evaluate the anti-metastatic efficacy of HJSS under inflammation-enhanced conditions, a peritoneal metastasis model was established using MKN-45 Luc gastric cancer cells (Figure 2A) [27,28]. As a potent pro-inflammatory agent, LPS is commonly used to simulate acute and chronic inflammatory states, enabling investigation of inflammation-related mechanisms and drug screening [29,30]. In this study, we employed LPS to induce chronic inflammation and evaluate the inhibitory effects of HJSS on GC metastasis under inflammatory conditions.

Longitudinal bioluminescence imaging revealed that LPS dramatically increased intraperitoneal tumor burden, as indicated by intensified fluorescent signals compared with controls. Notably, mice receiving HJSS exhibited a pronounced reduction in luminescent intensity, showing efficacy comparable to that observed in the 5-FU group (Figure 2B,C). At the experimental endpoint, inspection of peritoneal nodules confirmed significantly fewer and smaller metastatic lesions in the HJSS group relative to the LPS group, with an effect comparable to that observed in the 5-FU group (Figure 2D,E), further supporting the robust inhibitory effect of HJSS on LPS-driven peritoneal dissemination.

To assess systemic toxicity and organ injury, serum biochemical parameters were analyzed. LPS induced significant hepatic and renal impairment, evidenced by elevated ALT, AST, uric acid and creatinine levels. Remarkably, HJSS intervention effectively reversed these pathological alterations (Figure 2F–I), indicating potent hepatoprotective and renoprotective effects.

Histopathological examination further corroborated these findings. LPS and 5-FU groups displayed pronounced hepatic cellular swelling, necrotic foci, nuclear dissolution, fibrous hyperplasia, splenic nodular degeneration, and renal tubular atrophy with mesangial shrinkage. In contrast, tissues from HJSS-treated mice showed largely preserved hepatic, splenic, and renal architecture, with only mild cytoplasmic eosinophilia or tubular dilation (Figure 2J).

Collectively, these data demonstrate that HJSS robustly suppresses inflammation-driven gastric cancer metastasis and simultaneously protects major organs from LPS-induced injury, highlighting its dual functional advantages compared to conventional chemotherapy.

### 2.3. HJSS Attenuates LPS-Induced Migration, Invasion, and Cytoskeletal Remodeling in Gastric Cancer Cells

**Figure 3:**
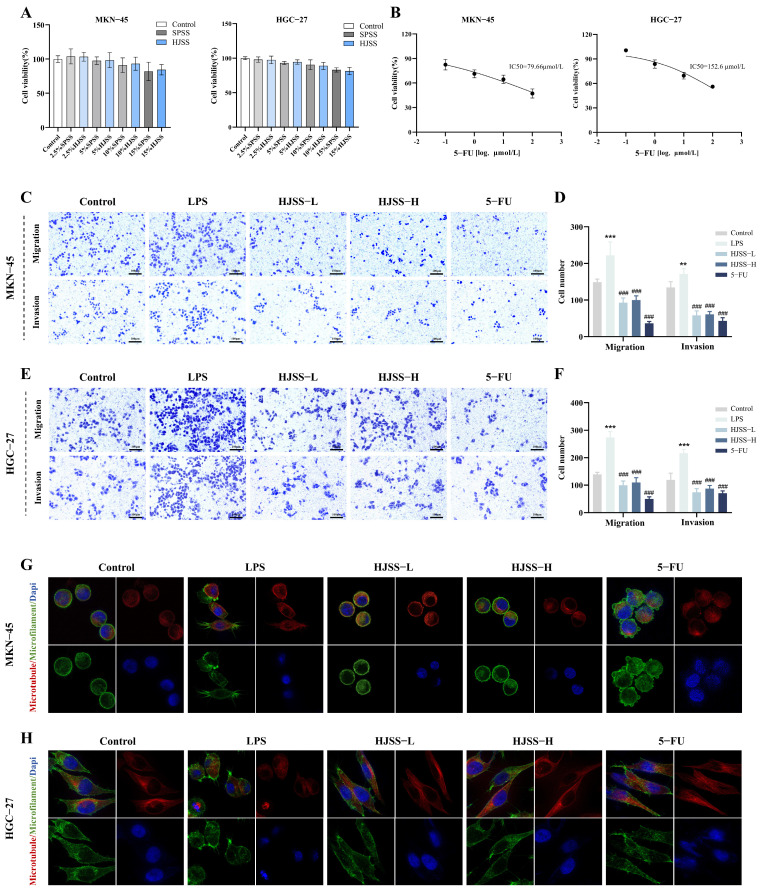
HJSS inhibits migration and invasion of gastric cancer cells and stabilizes cytoskeletal architecture. (**A**) Cytotoxicity of HJSS-containing serum in MKN-45 and HGC-27 cells assessed by CCK-8 assay. (**B**) Cytotoxicity of 5-FU in MKN-45 and HGC-27 cells assessed by CCK-8 assay. (**C**–**F**) Representative images and quantification of Transwell migration assays under different treatments in MKN-45 and HGC-27 cells. (**G**,**H**) Representative confocal images of microtubules and actin filaments (with DAPI nuclear staining) showing cytoskeletal organization in MKN-45 and HGC-27 cells following the indicated treatments (600×). Data are presented as mean ± SEM, *n* = 3. ** *p* < 0.01, *** *p* < 0.001, vs. Control group, ^###^ *p* < 0.001, vs. LPS group.

To determine the appropriate treatment concentrations for HJSS-containing serum, CCK-8 assays were performed using human GC cell lines. Results indicated that HJSS-containing serum did not significantly affect cell proliferation under the tested conditions, suggesting that its anti-metastatic effects are not primarily attributable to overt cytotoxicity (Figure 3A). 5-FU was employed as a positive control, and its half-maximal inhibitory concentrations (IC50) against MKN-45 and HGC-27 cells were determined by CCK-8 assays to establish optimal concentrations for subsequent experiments (Figure 3B).

We next evaluated the impact of HJSS on LPS-driven metastatic phenotypes. Transwell migration assays revealed that LPS markedly enhanced motility in both MKN-45 and HGC-27 cells, whereas HJSS significantly suppressed LPS-induced migration (Figure 3C,D). Similarly, LPS robustly increased invasive penetration through Matrigel-coated membranes, while HJSS treatment substantially reduced invasive cell numbers (Figure 3E,F). The inhibitory efficacy of HJSS on both migration and invasion was comparable to that of 5-FU.

Given the essential role of cytoskeletal remodeling in tumor cell motility [31,32], we further examined microtubule and actin filament organization by confocal microscopy (Figure 3G,H). LPS stimulation induced prominent cytoskeletal alterations, characterized by disorganized microtubule arrays, increased lamellipodia and filopodia formation, and disrupted actin filament networks, collectively indicating enhanced cellular plasticity. In contrast, HJSS treatment restored cytoskeletal integrity, showing more uniform microtubule distributions and organized F-actin structures with well-defined cell boundaries.

Together, these results demonstrate that HJSS attenuates inflammation-induced migration and invasion of GC cells and stabilizes cytoskeletal architecture, thereby limiting the acquisition of pro-metastatic phenotypes under inflammatory stimulation.

### 2.4. HJSS Reduces LPS-Induced Inflammatory Cytokine Expression and Macrophage Infiltration in Tumor Tissues

**Figure 4:**
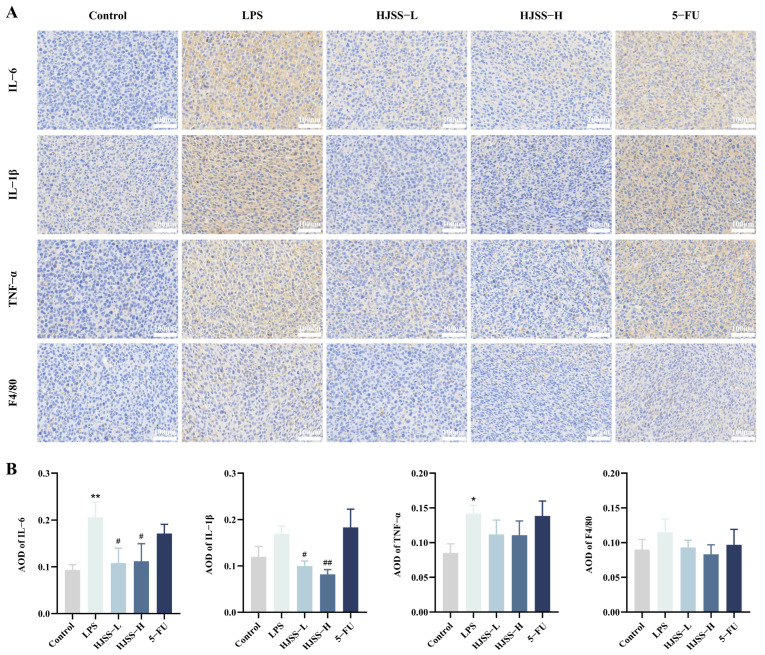
HJSS alleviates inflammatory responses and macrophage infiltration in tumor tissues. (**A**) Representative immunohistochemical staining of IL-6, IL-1β, TNF-α, and F4/80 in tumor tissues from each group. (**B**) Semi-quantitative analysis of IHC staining intensity/positive area for the indicated inflammatory markers. Data are presented as mean ± SEM, *n* = 3. * *p* < 0.05, ** *p* < 0.01, vs. Control group, ^#^ *p* < 0.05, ^##^ *p* < 0.01, vs. LPS group.

Given the prominent inflammatory component of the LPS-induced metastatic model, we next examined the effects of HJSS on key inflammatory mediators within tumor tissues. Immunohistochemical staining revealed that LPS markedly elevated the expression of pro-inflammatory cytokines IL-6, IL-1β, and TNF-α compared with the control group, indicating a strongly activated inflammatory microenvironment conducive to metastatic progression (Figure 4A,B). HJSS treatment significantly attenuated the expression of all three cytokines, restoring staining intensity toward baseline levels. These results are consistent with the systemic anti-inflammatory effects observed in serum analyses and demonstrate that HJSS effectively suppresses local inflammatory signaling within the tumor niche.

Because tumor-associated macrophages (TAMs) are key amplifiers of inflammation and are known to promote EMT, immune suppression, and metastasis in gastric cancer, we further examined macrophage infiltration using the F4/80 marker. LPS stimulation increased F4/80-positive cell accumulation, indicating enhanced macrophage recruitment and activation within tumor tissues (Figure 4A,B). HJSS reduced the abundance of infiltrating macrophages, suggesting its ability to mitigate LPS-induced inflammatory amplification loops mediated by TAMs.

Together, these findings indicate that HJSS not only restrains systemic inflammatory activation but also suppresses cytokine production and macrophage infiltration within the tumor microenvironment—factors that synergistically contribute to EMT and metastatic progression.

### 2.5. Network Pharmacology Reveals Potential Targets of HJSS in GC Metastasis

**Figure 5:**
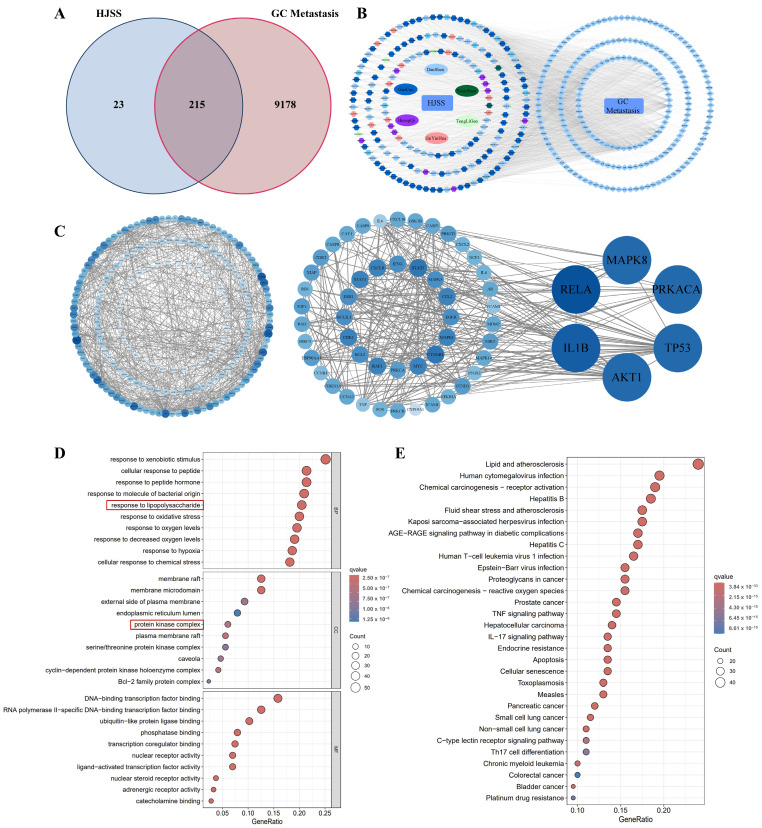
Network pharmacology identifies potential targets of HJSS against gastric cancer metastasis. (**A**) Venn diagram showing the overlap between HJSS-related targets and gastric cancer metastasis-associated genes. (**B**) Herb–compound–target network constructed in Cytoscape 3.8.0. (**C**) PPI network of overlapping targets generated from STRING and analyzed in Cytoscape/CytoNCA. Nodes represent proteins and edges represent protein–protein interactions. (**D**) GO enrichment analysis of overlapping targets. (**E**) KEGG pathway enrichment analysis of overlapping targets.

To elucidate the potential molecular basis of HJSS in GC metastasis, we first integrated HJSS-related targets with GC metastasis-associated genes. A total of 238 putative HJSS-related targets were retrieved after TCMSP-based compound screening and UniProt normalization, whereas 9393 gastric cancer metastasis-associated genes were collected from five public disease databases. Intersection analysis yielded 215 overlapping targets, which were considered potential therapeutic targets of HJSS against gastric cancer metastasis (Figure 5A). Construction of the herb–compound–target network revealed extensive connectivity between multiple HJSS-derived compounds and shared targets, supporting the multi-component and multi-target characteristics of the formula (Figure 5B). PPI network analysis and hub-gene identification showed that MAPK8, IL-1β, RELA, among others, occupy central positions within the network (Figure 5C). In the PPI network, nodes represent proteins and edges represent protein–protein interactions. Topological parameters, including degree, betweenness centrality, and closeness centrality, were used to identify hub targets with potentially important regulatory roles. These core nodes are closely linked to inflammatory signaling, stress responses, and tumor progression.

GO and KEGG enrichment analyses demonstrated that the 215 overlapping targets are mainly involved in biological processes such as responses to external stimuli, regulation of oxidative stress, and defense against bacterial infection, as well as multiple cancer-related pathways (Figure 5D,E). Notably, among enriched protein kinase families, serine/threonine kinases—particularly members of the MAPK family—were predominant, indicating that MAPK-related kinase cascades may represent an important regulatory axis through which HJSS modulates GC metastasis. These findings provide a systems-level rationale for our subsequent focus on MAPK family members, especially JNK, in the mechanistic validation experiments.

### 2.6. Transcriptome Sequencing Reveals That HJSS Attenuates LPS-Activated Serine/Threonine Kinase Signaling and Cadherin-Related Pathways

**Figure 6:**
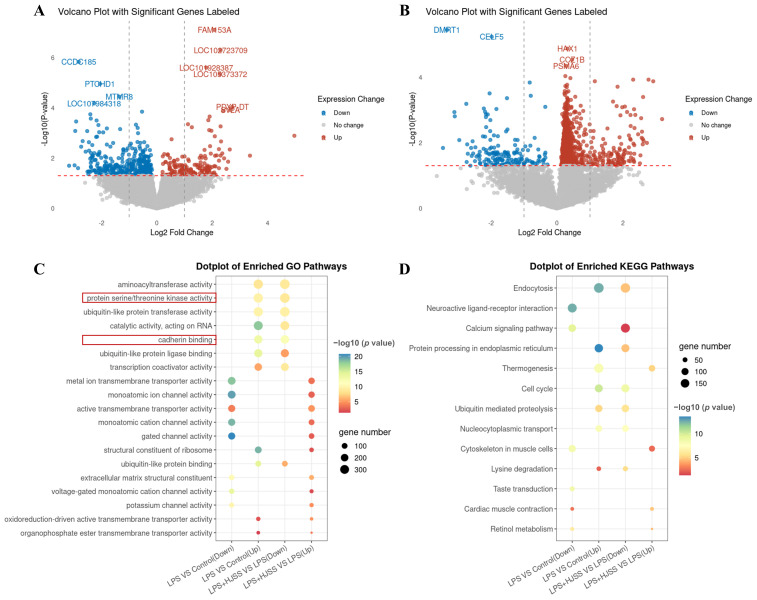
Transcriptomic analysis reveals biological processes and pathways modulated by HJSS in the inflammatory gastric cancer model. (**A**) Volcano plot of differentially expressed genes (DEGs) between the Control group and the LPS group. (**B**) Volcano plot of DEGs between the LPS group and the HJSS group. (**C**) GO enrichment analysis of DEGs. (**D**) KEGG pathway enrichment analysis of DEGs.

To complement the prediction-based network pharmacology analysis with an unbiased in vivo molecular readout, transcriptome sequencing was performed to identify gene-expression programs altered by LPS stimulation and reversed by HJSS. The volcano plot showed that LPS induced a substantial number of differentially expressed genes (DEGs) compared with the control group (Figure 6A). HJSS treatment resulted in a distinct DEG profile relative to the LPS group (Figure 6B).

GO enrichment analysis revealed significant enrichment of serine/threonine kinase activity among LPS-responsive genes (Figure 6C). Given that JNK (MAPK8) is a serine/threonine kinase within the MAPK family, this enrichment result is compatible with, but not by itself specific for, the involvement of MAPK-related signaling, in line with the network pharmacology analysis. In addition, genes associated with cadherin binding were also markedly enriched. Because cadherin-mediated adhesion is central to epithelial–mesenchymal transition (EMT), this transcriptional pattern is consistent with the migratory and invasive phenotypes observed in vitro. KEGG pathway analysis further identified enrichment of inflammation- and cancer-related pathways (Figure 6D), suggesting that kinase regulation and adhesion remodeling are key molecular responses to LPS stimulation.

Together, the transcriptomic data provide in vivo molecular support for the network pharmacology prediction and indicate that HJSS modulates inflammation-associated kinase signaling and EMT-related adhesion remodeling, thereby justifying the subsequent focus on the JNK–EMT axis.

### 2.7. HJSS Reverses LPS-Induced EMT Marker Changes in Tumor Tissues and MKN-45 Cells

To investigate whether the anti-metastatic effects of HJSS are associated with changes in EMT status, we examined representative epithelial and mesenchymal markers in tumor tissues and MKN-45 cells.

**Figure 7:**
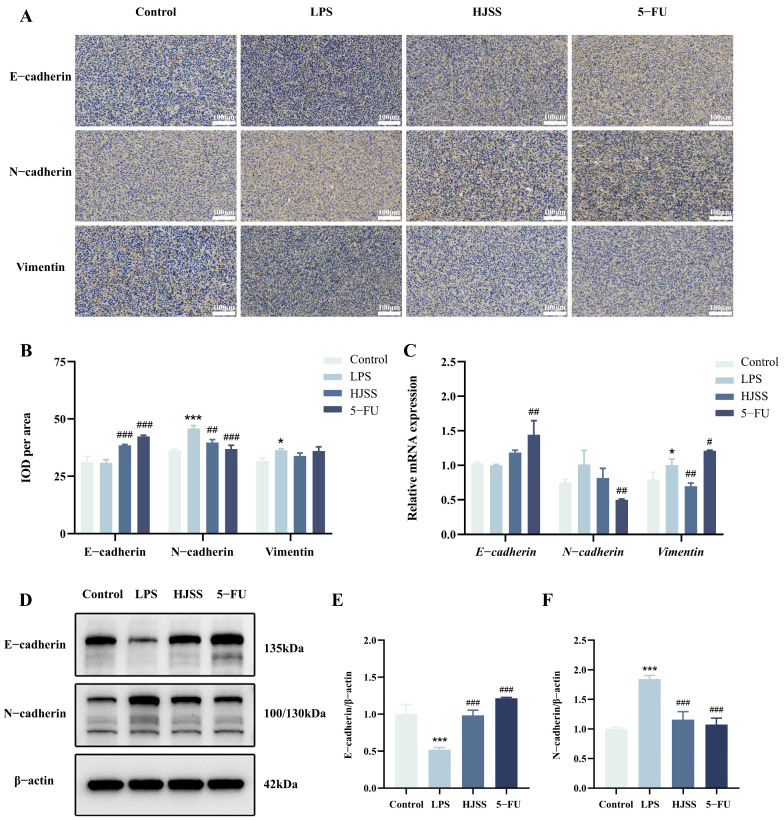
HJSS inhibits inflammation-induced EMT progression in vivo and in vitro. (**A**) Representative immunohistochemical staining of E-cadherin, N-cadherin, and Vimentin in tumor tissues. (**B**) Semi-quantitative analysis of EMT-related IHC staining. (**C**) qPCR analysis of E-cadherin, N-cadherin, and Vimentin mRNA levels in MKN-45 cells under different treatments. (**D**) Representative Western blot analysis of E-cadherin and N-cadherin protein levels in MKN-45 cells. (**E**,**F**) Densitometric quantification of Western blot results. Data are presented as mean ± SEM, *n* = 3. * *p* < 0.05, *** *p* < 0.001, vs. Control group, ^#^ *p* < 0.05, ^##^ *p* < 0.01, ^###^ *p* < 0.001, vs. LPS group.

Immunohistochemical staining showed that LPS markedly reduced the epithelial marker E-cadherin and increased the mesenchymal markers N-cadherin and Vimentin in tumor tissues compared with the control group, indicating that LPS promotes EMT in vivo. Treatment with HJSS significantly restored E-cadherin expression and decreased N-cadherin and Vimentin levels, suggesting that HJSS effectively reverses inflammation-induced EMT activation in the tumor microenvironment (Figure 7A,B).

At the cellular level, qPCR analysis in MKN-45 cells revealed that LPS downregulated E-cadherin mRNA while upregulating N-cadherin and Vimentin transcripts, consistent with an EMT-like transcriptional shift. HJSS treatment significantly attenuated these changes, increasing E-cadherin mRNA and reducing N-cadherin and Vimentin mRNA expression (Figure 7C). Western blot analysis further confirmed these trends at the protein level (Figure 7D–F). LPS decreased E-cadherin and increased N-cadherin protein expression in MKN-45 cells, whereas HJSS partially reversed these alterations, restoring an expression pattern more characteristic of an epithelial phenotype.

Taken together, these data demonstrate that HJSS counteracts LPS-induced EMT marker switching in both tumor tissues and MKN-45 cells, supporting a key role for EMT inhibition in the anti-metastatic effects of HJSS. In addition, RT-qPCR analysis showed that HJSS also attenuated the LPS-induced upregulation of EMT-related transcription factors, including Snail, Slug, Twist, and ZEB1, further supporting its inhibitory effect on EMT-associated transcriptional programs (Figure S1A).

### 2.8. HJSS Suppresses LPS-Induced Nuclear Accumulation and Phosphorylation of JNK Without Altering Total JNK Levels

**Figure 8:**
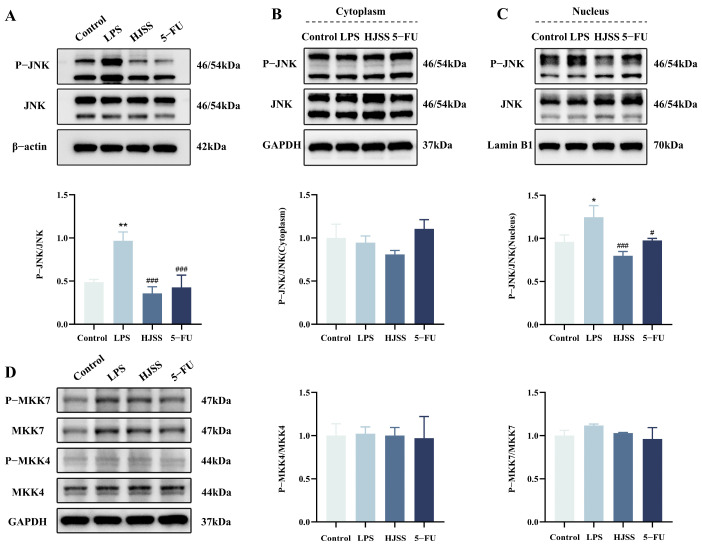
HJSS suppresses inflammation-induced JNK activation and nuclear accumulation of p-JNK. (**A**) Representative Western blot analysis of total JNK and phosphorylated JNK (p-JNK) in MKN-45 cells. (**B**) Western blot analysis of JNK and p-JNK in cytoplasmic fractions (GAPDH as loading control). (**C**) Western blot analysis of JNK and p-JNK in nuclear fractions (Lamin B1 as loading control). (**D**) Western blot analysis of MKK4/MKK7 and their phosphorylation levels in MKN-45 cells. Corresponding densitometric quantification is shown below each blot. Data are presented as mean ± SEM, *n* = 3. * *p* < 0.05, ** *p* < 0.01, vs. Control group, ^#^ *p* < 0.05, ^###^ *p* < 0.001, vs. LPS group.

To clarify how JNK signaling contributes to the anti-metastatic effects of HJSS, we first examined both total and phosphorylated JNK (p-JNK) in whole-cell lysates of MKN-45 cells. Western blotting showed that LPS stimulation did not markedly alter total JNK expression, but significantly increased the phosphorylation level of JNK, indicating activation of the pathway. HJSS treatment markedly reduced LPS-induced p-JNK while leaving total JNK levels essentially unchanged (Figure 8A).

Because JNK activation depends on phosphorylation and nuclear translocation, we next performed nuclear–cytoplasmic fractionation. In the cytoplasmic fraction, both total JNK and phosphorylated JNK (p-JNK) remained largely unchanged among the treatment groups (Figure 8B). In contrast, the nuclear fraction showed a pronounced increase in nuclear p-JNK following LPS stimulation, demonstrating robust activation and nuclear accumulation of JNK under inflammatory conditions (Figure 8C). Importantly, HJSS markedly reduced nuclear p-JNK levels, indicating that HJSS suppresses JNK activation, as reflected by a marked reduction in nuclear p-JNK accumulation under inflammatory conditions.

Together, these results demonstrate that HJSS suppresses LPS-triggered JNK activation by reducing its nuclear phosphorylation and accumulation.

### 2.9. HJSS Does Not Alter Upstream Kinases MKK4/7, and CETSA Plus Molecular Docking Support Potential Interactions Between HJSS-Derived Constituents and JNK

To determine whether the inhibitory effect of HJSS on JNK activation is mediated by upstream MAPKs, we examined the expression of MKK4 and MKK7, the canonical kinases responsible for JNK phosphorylation. Western blot analysis revealed that LPS stimulation did not significantly alter MKK4 or MKK7 protein levels, and HJSS treatment also showed no detectable impact on these kinases (Figure 8D). These findings suggest that the modulation of JNK activation by HJSS is unlikely to result from regulation of upstream MKK4/7 expression.

**Figure 9:**
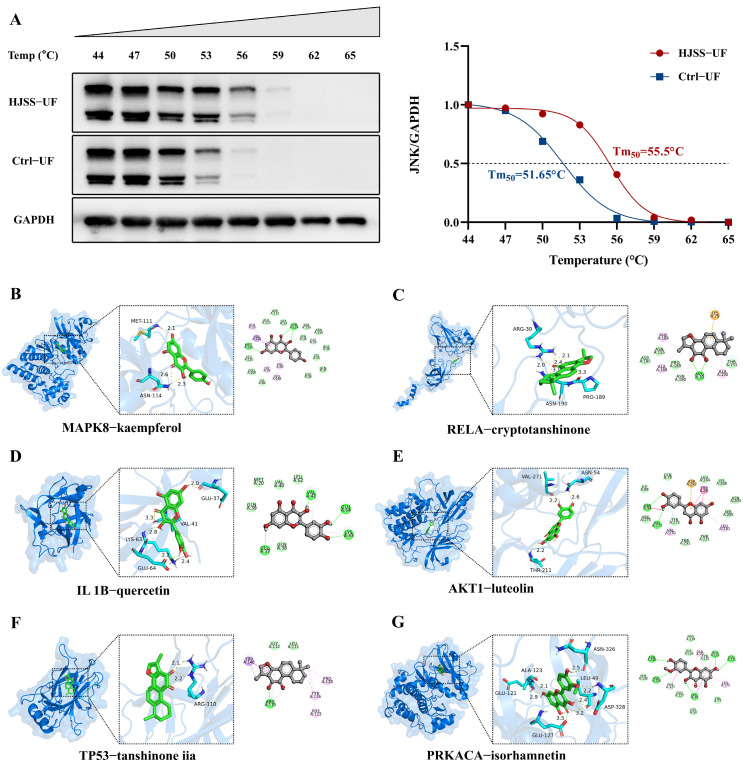
CETSA and molecular docking support potential target engagement by HJSS-derived constituents. (**A**) CETSA was performed to assess changes in JNK thermal stability in MKN-45 cells treated with HJSS ultrafiltrates. (**B**–**G**) Representative docking models of selected HJSS-derived compounds with the corresponding hub targets identified by network pharmacology, including MAPK8–kaempferol, RELA–cryptotanshinone, IL1B–quercetin, AKT1–luteolin, TP53–tanshinone IIA, and PRKACA–isorhamnetin.

Given the unchanged levels of upstream kinases, we hypothesized that low-molecular-weight constituents within HJSS may interact with JNK. To test this hypothesis, we performed a cellular thermal shift assay (CETSA) using ultrafiltration-purified low-molecular-weight fractions of HJSS. CETSA results demonstrated a clear upward shift in the thermal stability of JNK in the presence of HJSS fractions (Figure 9A), indicating that one or more small-molecule components in HJSS may engage JNK and alter its thermal stability, thereby stabilizing the protein structure.

To further explore candidate compound–target relationships, molecular docking was conducted for six representative bioactive compounds against the corresponding hub targets identified by network pharmacology. Among these, kaempferol showed favorable predicted binding to MAPK8/JNK, whereas the other representative compounds were docked to RELA, IL1B, AKT1, TP53, and PRKACA, respectively (Figure 9B–G), suggesting stable ligand–protein interactions at functionally relevant binding pockets. These computational predictions support the biochemical evidence from CETSA. To provide preliminary functional support for a representative constituent, luteolin was further examined in MKN-45 cells and was found to reduce LPS-induced JNK phosphorylation, consistent with the effect of HJSS on JNK activation (Figure S1B,C).

Collectively, these findings suggest that HJSS modulates JNK activation not through overt changes in MKK4/7 expression, but potentially through interactions between HJSS-derived constituents and JNK or related signaling targets, and may thereby contribute to reduced JNK phosphorylation and nuclear accumulation. This offers a plausible mechanistic framework for how HJSS suppresses JNK-associated EMT and metastatic progression.

### 2.10. JNK Activation with Anisomycin Partially Reverses the Anti-EMT and Anti-Metastatic Effects of HJSS

To further assess whether the inhibitory effects of HJSS on EMT and metastasis involve the JNK/MAPK pathway, we employed anisomycin, a well-characterized JNK activator, to pharmacologically reactivate JNK signaling in HJSS-treated gastric cancer cells.

**Figure 10:**
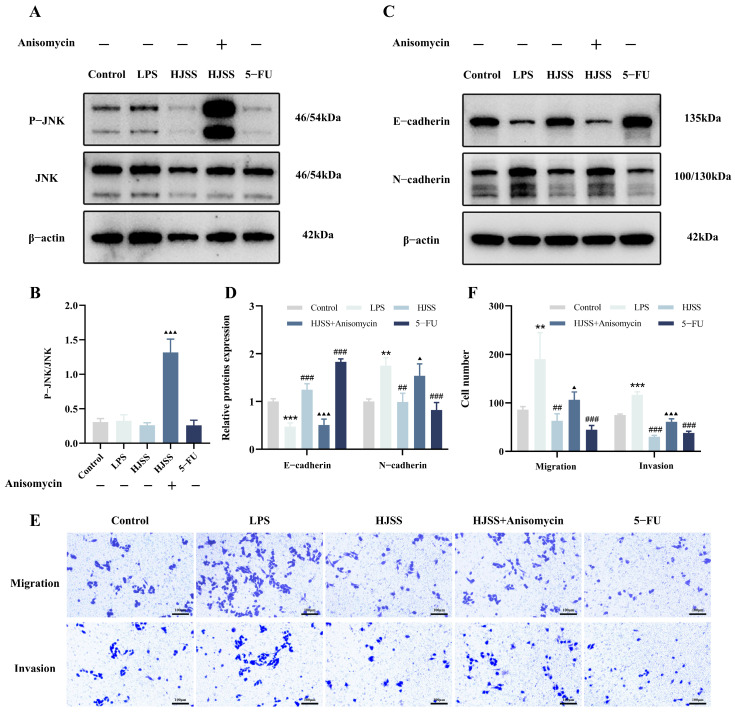
JNK activation by anisomycin partially reverses the anti-EMT and anti-metastatic effects of HJSS. (**A**,**B**) Western blot analysis and quantification of JNK phosphorylation in MKN-45 cells treated with anisomycin. (**C**,**D**) Western blot analysis and quantification of EMT markers (E-cadherin and N-cadherin) following anisomycin treatment. (**E**) Representative images of Transwell migration and invasion assays. (**F**) Quantification of migrated/invaded cells under the indicated treatments. Data are presented as mean ± SEM, *n* = 3. ** *p* < 0.01, *** *p* < 0.001, vs. Control group, ^##^ *p* < 0.01, ^###^ *p* < 0.001, vs. LPS group, ^▲^ *p* < 0.05, ^▲▲▲^ *p* < 0.001, vs. HJSS group.

Western blot analysis showed that anisomycin markedly increased JNK phosphorylation (p-JNK) in cells pretreated with HJSS, while total JNK levels remained essentially unchanged (Figure 10A,B). Concomitantly, anisomycin decreased the expression of the epithelial marker E-cadherin and increased the mesenchymal marker N-cadherin in the presence of HJSS (Figure 10C,D). These changes indicate that reactivation of JNK signaling can partially reverse the EMT-suppressive effects of HJSS at the molecular level.

Functionally, Transwell migration and invasion assays further supported this observation. In HJSS-treated gastric cancer cells, anisomycin significantly enhanced both migratory and invasive capacities (Figure 10E,F), thereby weakening the anti-metastatic effects of HJSS under inflammatory conditions. Although anisomycin did not fully restore the highly motile phenotype observed in LPS-stimulated cells without HJSS, the consistent reversal of EMT markers and cell motility strongly supports a JNK-dependent component in the action of HJSS. Consistent with this interpretation, treatment with the JNK inhibitor SP600125 further supported the involvement of JNK signaling by partially reproducing the EMT-related changes associated with JNK suppression, as reflected by E-cadherin and N-cadherin expression patterns (Figure S1D–F).

**Figure 11:**
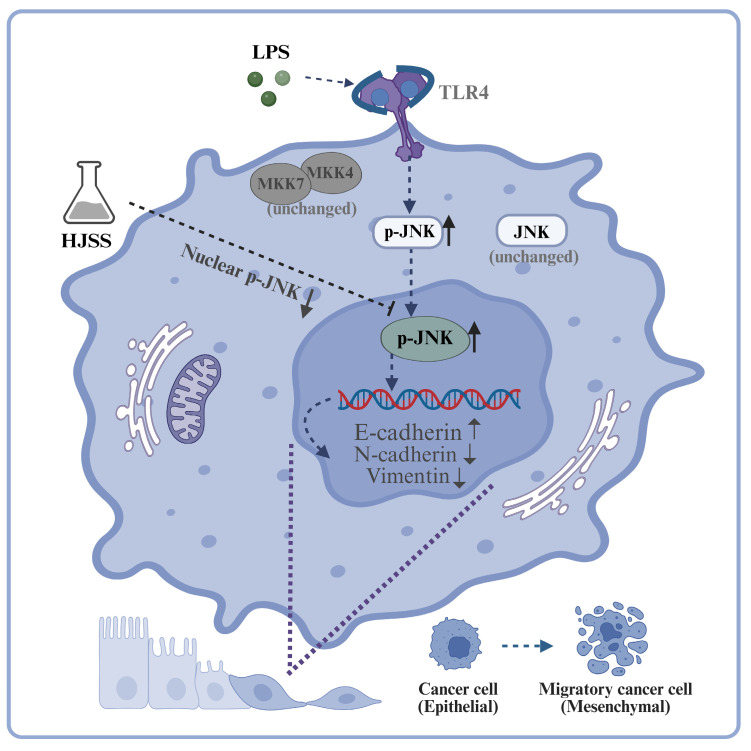
Proposed model illustrating how HJSS suppresses EMT and metastasis via the JNK/MAPK pathway under inflammatory stress. LPS triggers inflammatory signaling and increases JNK phosphorylation, promoting nuclear accumulation of p-JNK and activation of EMT-associated transcriptional programs, thereby facilitating migratory and invasive phenotypes. HJSS suppresses JNK activation and prominently attenuates nuclear p-JNK accumulation, leading to inhibition of EMT progression and metastatic dissemination.

Taken together with the preceding data on JNK phosphorylation, nuclear accumulation, and EMT marker regulation, these findings support the conclusion that HJSS suppresses gastric cancer cell migration and invasion, at least in part, through modulation of the JNK/MAPK pathway and associated EMT-related changes. A schematic model summarizing this proposed mechanism is shown in Figure 11.

## 3. Discussion

Chronic inflammation is now recognized as a fundamental enabling factor of cancer progression, including gastric cancer (GC), by reshaping the tumor microenvironment and sustaining malignant cell plasticity [33,34]. In this context, EMT has emerged as a key biological program linking inflammatory cues to metastatic dissemination: epithelial cells gradually lose polarity and cell–cell adhesion and acquire mesenchymal traits, a process coordinated by multiple signaling pathways and transcriptional regulators. EMT is not a simple on–off switch but a dynamic, reversible continuum that allows carcinoma cells to occupy intermediate hybrid states with high metastatic potential [35,36]. Against this background, our LPS-based inflammatory model provided a tractable experimental setting to examine how HJSS modulates inflammation-associated EMT and metastasis [37].

To complement the prediction-based network pharmacology analysis with an unbiased in vivo molecular readout, we further performed transcriptome sequencing to identify gene-expression programs altered by LPS stimulation and modulated by HJSS. This integrative approach allowed us to assess whether the pathways highlighted by network pharmacology were also supported by experimentally derived transcriptional changes.

In the present study, HJSS attenuated LPS-enhanced metastatic behavior in vivo and in vitro while reversing EMT marker switching at both mRNA and protein levels. These phenotypic changes were accompanied by transcriptomic enrichment of cadherin-binding and cytoskeleton-related pathways, in line with the established role of EMT in adhesion remodeling, motility acquisition, and metastatic colonization. Together, these findings support the view that HJSS interferes with an EMT-centered metastatic program amplified by inflammation, rather than acting solely as a cytotoxic agent.

Our data further indicate that the JNK/MAPK axis is an important component of this regulatory effect. JNK is a stress-activated kinase that integrates inflammatory, oxidative, and microenvironmental stimuli and cooperates with transcription factors such as AP-1 and Snail family proteins to promote EMT and invasion. Although total JNK expression remained relatively stable in our system, LPS robustly increased JNK phosphorylation and nuclear accumulation, consistent with JNK activation under inflammatory stress. HJSS reduced nuclear p-JNK without substantially altering cytoplasmic JNK, indicating that it primarily regulates the activation state and subcellular dynamics of JNK rather than its overall abundance. The ability of anisomycin, a well-characterized activator of JNK signaling [38,39], to partially restore p-JNK levels, reverse E-/N-cadherin changes and enhance migration/invasion in HJSS-treated cells provides functional evidence that JNK contributes to the anti-EMT and anti-metastatic actions of HJSS.

Interestingly, the canonical upstream kinases MKK4 and MKK7 did not show obvious changes at the total protein level after LPS or HJSS treatment. While this does not exclude regulation at the level of phosphorylation or scaffold interactions, it suggests that the modulation of JNK by HJSS is unlikely to be mediated simply by downregulating MAP2K expression [40]. In our study, CETSA indicated that HJSS-derived low-molecular-weight fractions increase the thermal stability of JNK, and molecular docking showed that selected constituents of HJSS could adopt favorable binding conformations with JNK or other hub targets identified by network pharmacology. Coupled with prior reports that TCM-derived compounds such as astragaloside IV and tanshinones modulate MAPK and NF-κB signaling [41,42], these observations raise the possibility that HJSS regulates JNK at least partly through constituent-mediated effects on JNK target engagement or conformational state.

Our findings are also consistent with accumulating evidence that TCM formulas and natural products can restrain metastasis and EMT in GC. For example, β-elemene was reported to inhibit EMT and metastatic capacity of multidrug-resistant gastric cancer cells by modulating EGFR–ERK/AKT signaling [43], and Compound Kushen Injection suppressed EMT in GC through regulation of the TNF signaling pathway and adhesion-related molecules [44]. Other studies using network pharmacology combined with experimental validation have shown that compound prescriptions such as Weikang Keli or Huangqi Fuling Decoction exert anti-GC effects by converging on MAPK, apoptosis, or autophagy-related pathways [45,46]. Within this growing body of work, our study adds mechanistic support for HJSS as another formula capable of regulating an inflammation–EMT–kinase axis—in this case, by dampening JNK activation and nuclear p-JNK accumulation in an LPS-driven EMT context. An additional strength of the present study is that the pharmacological observations were supported by chemical profiling of HJSS. UPLC-QTOF-MS analysis identified multiple representative constituents spanning flavonoids, phenolic acids, diterpenoid quinones, and saponins, while several docking-related compounds, including luteolin, quercetin, kaempferol, cryptotanshinone, and isorhamnetin, were directly detected in the formula. These findings reduce the “black-box” nature of the herbal preparation and provide a preliminary chemical basis for the observed modulation of JNK-associated signaling. Given the multi-component nature of HJSS, it is likely that the biological effects arise from coordinated actions of multiple constituents rather than from a single dominant compound, which is also consistent with current views on multi-component traditional Chinese medicine in cancer therapy.

At the same time, several issues merit cautious interpretation. First, anisomycin only partially reversed the anti-EMT and anti-metastatic effects of HJSS, suggesting that JNK is an important but not exclusive effector. Other inflammation-related pathways, including NF-κB, or IL-6/STAT3, have well-documented roles in EMT and microenvironmental remodeling [47,48], and HJSS may exert additional influences on these networks. Second, CETSA and docking support a model of potential modulation of JNK by HJSS components but do not by themselves identify precise binding sites or active compounds. Further fractionation, binding assays, and structure–activity relationship studies will be necessary to move from a multi-component mixture to defined molecular entities. Third, while LPS is widely used to establish inflammatory models, it does not capture all clinically relevant inflammatory drivers of GC, such as chronic *Helicobacter pylori* infection or peritoneal dissemination in advanced disease [49,50]. Validation in additional models, including H. pylori-associated precancerous lesions and peritoneal metastasis systems, will be important for translation.

Collectively, HJSS suppresses LPS-driven metastatic traits in gastric cancer and mitigates inflammation-associated EMT. Our data highlight nuclear p-JNK as a key signaling event modulated by HJSS within the JNK/MAPK axis. This work supports HJSS as a multi-target intervention for inflammation-associated gastric cancer progression and motivates further efforts to define the contribution of specific HJSS-derived constituents in clinically relevant models.

## 4. Materials and Methods

### 4.1. Preparation of HJSS Granules and HJSS-Containing Serum

HJSS granules was provided by Zhejiang Chinese Medical University Pharmaceutical Co., Ltd. (Hangzhou, China). The herbal extract was prepared by standardized industrial granules and concentration procedures and stored at 4 °C until use. For the preparation of HJSS-containing serum, male Sprague-Dawley rats (180–220 g) were randomly divided into an HJSS group and a vehicle group. Rats received oral gavage of HJSS or an equal volume of distilled water twice daily for 3 days. One hour after the final administration, rats were anesthetized and blood was collected from the cardiac apex. Serum was separated by centrifugation, heat-inactivated at 56 °C for 30 min, filtered through a 0.22 μm membrane, aliquoted, and stored at −80 °C. All procedures were approved by the Ethics Committee of Zhejiang Chinese Medical University (Ethics Approval No. 20220418-27; Animal License SYXK2021-0012).

### 4.2. UPLC-QTOF-MS Analysis of HJSS

The chemical profile of HJSS was analyzed using ultra-performance liquid chromatography coupled with quadrupole time-of-flight mass spectrometry (UPLC–QTOF–MS). HJSS samples were filtered prior to analysis and separated on a reversed-phase C18 column using a gradient elution system. Mass spectrometric data were acquired in both positive and negative electrospray ionization modes, and base peak chromatograms (BPCs) were generated to characterize the overall chemical fingerprint of HJSS. Putative compounds were tentatively identified based on accurate mass and MS/MS fragmentation information, providing the chemical basis for subsequent network pharmacology analysis. Compound annotation was further supported by integrating precursor ion information, retention time, mass error, and characteristic MS/MS fragment ions using the LuMet-TCM database and reference spectral information where available. Representative annotation evidence for major constituents is summarized in Table S3.

### 4.3. Cell Culture and Treatment Groups

Human gastric cancer cell lines MKN-45, MKN-45-Luc, and HGC-27 were obtained from the Zhejiang Academy of Traditional Chinese Medicine (Hangzhou, China). Cells were cultured in RPMI-1640 medium supplemented with 10% fetal bovine serum (SA201.02, CellMax, Beijing, China) and 1% penicillin-streptomycin at 37 °C in a humidified incubator with 5% CO_2_. For in vitro assays, cells were assigned to the following groups: Control (control serum); LPS (1 μg/mL lipopolysaccharide; L2880, SIGMA, Billerica, USA); HJSS (low/high); 5-FU (HY-90006, MCE, Monmouth Junction, NJ, USA); and, where indicated, additional treatment groups involving luteolin, anisomycin, or SP600125 for mechanistic validation. Treatment durations (24–48 h) were adjusted according to the specific assay.

### 4.4. LPS-Enhanced Peritoneal Metastasis Model of Gastric Cancer

Male BALB/c nude mice (4–5 weeks old, 18–20 g) were purchased from Shanghai Slack Laboratory Animal Co., Ltd. (Shanghai, China) and housed under SPF conditions (temperature: 18–25 °C, humidity: 50–60%, 12 h light/dark cycle) in the Animal Center of Zhejiang Chinese Medical University. Mice were acclimatized for at least 1 week before the start of the experiment. Sample sizes were determined based on our previous experience with this peritoneal metastasis model and preliminary experiments, taking into account feasibility, expected biological variation, and ethical considerations to minimize animal use. A total of 25 mice were used, with *n* = 5 per group. A gastric cancer peritoneal metastasis model was established by intraperitoneal injection of 0.2 mL of MKN-45-Luc cell suspension (2 × 10^7^ cells/mL in sterile PBS). After tumor cell implantation, mice were randomly assigned to different experimental groups using a random number method: Control, LPS, HJSS (L/H), and 5-FU. Animals from different groups were housed under the same environmental conditions and handled in parallel to minimize potential confounding factors. LPS (0.5 mg/kg) was administered to induce systemic inflammation. HJSS granules and 5-FU were administered by oral gavage or intraperitoneal injection, respectively, according to the experimental design. Tumor burden and metastatic dissemination were monitored by in vivo bioluminescence imaging after intraperitoneal injection of D-luciferin (122799, PerkinElmer, Waltham, MA, USA) using an imaging system. The primary outcome measure for the animal study was intraperitoneal tumor burden assessed by bioluminescence imaging and metastatic nodule counts. No predefined exclusion criteria were applied during the animal experiments or data analysis unless technical failure occurred. No animals or data points were excluded from the final analysis. Animals were monitored daily for general condition, body weight, and signs of distress throughout the study. No unexpected adverse events occurred. Humane endpoints were predefined, and animals showing severe distress, marked weight loss, or impaired mobility would be euthanized immediately.

### 4.5. Histology, Blood Biochemistry, and Immunohistochemistry

Fresh tumor and organ tissues were fixed in 4% paraformaldehyde, dehydrated, embedded in paraffin, and sectioned at 4 μm. Hematoxylin-eosin staining was performed to evaluate general histology and metastatic lesions. Blood samples were centrifuged to obtain serum, and liver and kidney function indices, as well as routine hematological parameters, were measured using an automated biochemical and hematology analyzer according to the manufacturer’s instructions. For immunohistochemistry, deparaffinized sections were subjected to antigen retrieval, endogenous peroxidase blocking, and non-specific blocking, followed by incubation with primary antibodies against EMT- and inflammation-related markers (antibody information is provided in Table S4). After incubation with HRP-conjugated secondary antibodies and DAB development, sections were counterstained with hematoxylin, imaged under a light microscope, and quantified using ImageJ 1.54p. Investigators performing histological evaluation and image quantification were blinded to group allocation.

### 4.6. Cell Viability Assay

Cell viability was determined using a Cell Counting Kit-8 (C0037, Beyotime, Shanghai, China). Briefly, cells were seeded into 96-well plates at 5 × 10^3^ cells/well and allowed to adhere overnight, then treated with HJSS-containing serum, LPS, 5-FU, or their combinations for 24–48 h as indicated. At the end of treatment, 10 μL of CCK-8 solution was added to each well and incubated at 37 °C for 1–2 h. Absorbance at 450 nm was measured using a microplate reader, and relative viability was calculated by normalizing to the Control group.

### 4.7. Transwell Migration and Invasion Assays

Cell migration and invasion were evaluated using Transwell chambers with 8 μm pore size (CLS3422, Corning, Corning, NY, USA). For migration assays, the upper chamber was pre-equilibrated with serum-free medium, and treated cells were resuspended in serum-free medium containing the respective treatments and seeded into the upper chamber. For invasion assays, the upper chamber was precoated with Matrigel (356234, BD Biosciences, Franklin Lakes, NJ, USA) diluted in serum-free medium and incubated at 37 °C for gel formation before cell seeding. The lower chamber was filled with medium containing 20% FBS as a chemoattractant. After 48 h incubation, cells on the upper surface of the membrane were gently removed, and cells that migrated or invaded to the lower surface were fixed with 4% paraformaldehyde and stained with 0.1% crystal violet. Stained cells were photographed under a microscope (AE2000, Motic, Xiamen, China) and counted in several random fields using ImageJ 1.54p.

### 4.8. Cytoskeleton and Immunofluorescence Staining

For cytoskeletal and p-JNK localization analysis, sterile glass coverslips were coated with poly-L-lysine (C0313, Beyotime), and treated cells were seeded and allowed to attach. Cells were then fixed with 4% paraformaldehyde, permeabilized with 0.3% Triton X-100, and blocked with 5% BSA. For cytoskeleton staining, cells were incubated with Tubulin-Tracker Red (C1050, Beyotime) and Actin-Tracker Green (C2201S, Beyotime) in secondary antibody diluent, protected from light, for 1 h at room temperature. Nuclei were counterstained with DAPI, and fluorescence images were acquired using a confocal laser scanning microscope (Zeiss LSM800, Oberkochen, BW, Germany).

### 4.9. Nuclear-Cytoplasmic Extraction and Western Blot

Nuclear and cytoplasmic proteins were extracted using a commercial separation kit (P0027, Beyotime). Lysates were prepared in RIPA buffer containing protease/phosphatase inhibitors. Protein concentrations were quantified using a BCA kit (P0012, Beyotime). Equal protein amounts were separated by SDS-PAGE and transferred onto PVDF membranes. Membranes were blocked using a protein-free rapid blocking buffer and incubated overnight at 4 °C with primary antibodies. After incubation with HRP-conjugated secondary antibodies, bands were visualized using an ECL detection system (P0018S, Beyotime). Lamin B1 and GAPDH served as markers for nuclear and cytoplasmic fractions, respectively.

### 4.10. Quantitative Real-Time PCR

Total RNA from cells was extracted using TRIzol reagent following the manufacturer’s protocol. RNA purity and concentration were determined spectrophotometrically, and equal amounts of RNA were reverse-transcribed into cDNA using a commercial reverse transcription kit (RC112-01, Vazyme, Nanjing, China). Quantitative real-time PCR was performed with SYBR Green Master Mix on a real-time PCR system. The 18S rRNA gene was used as the internal reference. Relative expression levels were calculated by the 2^−ΔΔCt^ method. Primer sequences are listed in Table S5.

### 4.11. Transcriptome Sequencing and Bioinformatic Analysis

Tumor tissues from the Control, LPS, and HJSS groups were collected for RNA sequencing. Total RNA was extracted and submitted to OE Biotech Co., Ltd. (Shanghai, China) for library construction and sequencing. Sequencing was performed on an Illumina platform. Differentially expressed genes were identified using |log_2_FC| > 1 and *p* < 0.05 thresholds. GO and KEGG enrichment analyses were conducted using RStudio 2024.04.2 to identify biological pathways affected by LPS stimulation and HJSS intervention. Volcano plots and enrichment maps were generated using standard bioinformatic procedures.

### 4.12. Network Pharmacology and Molecular Docking

Active compounds of HJSS were initially retrieved from the Traditional Chinese Medicine Systems Pharmacology Database and Analysis Platform (TCMSP, https://www.tcmsp-e.com/tcmsp.php (accessed on 12 December 2024)). ADME-related screening was performed using oral bioavailability (OB > 30%) and drug-likeness (DL > 0.18) as filtering criteria. The targets corresponding to the active compounds of the seven herbs in HJSS were integrated and deduplicated. Target proteins were then standardized in the UniProt database, https://www.uniprot.org/ (accessed on 12 December 2024), and only reviewed human targets were retained for gene name normalization. Disease-related targets were collected using ‘Gastric Cancer Metastasis’ as the keyword from five public databases, including GeneCards, OMIM, DrugBank, PharmGKB, and TTD. After merging and deduplication, the resulting dataset was used as the gastric cancer metastasis-related target set. The overlap between HJSS-related targets and gastric cancer metastasis-associated targets was identified using Venny 2.1.0, and the intersecting genes were considered potential targets of HJSS against gastric cancer metastasis. These targets were imported into Cytoscape 3.8.0 to construct and visualize the herb–compound–target network. For protein–protein interaction (PPI) analysis, the intersecting targets were submitted to the STRING database, with species restricted to Homo sapiens, a confidence score > 0.9, and isolated nodes removed. The resulting PPI network was imported into Cytoscape and analyzed using the CytoNCA plugin to identify core targets through stepwise topological screening. For functional annotation, the intersecting targets were converted to gene IDs in RStudio 2024.04.2, followed by Gene Ontology (GO) and Kyoto Encyclopedia of Genes and Genomes (KEGG) enrichment analyses. A q value < 0.05 was used as the significance threshold, and the top enriched GO terms and KEGG pathways were visualized.

For molecular docking, target protein sequences were retrieved from UniProtKB and structurally modeled using SWISS-MODEL. Ligand structures were downloaded from public small-molecule databases and energy-minimized in Discovery Studio. Protein structures were preprocessed in PyMOL 3.1 by removing water molecules and redundant ligands, followed by hydrogen addition and charge assignment. Ligands were also prepared by hydrogen addition, charge assignment, and torsion-bond checking. Receptor and ligand files were converted into PDBQT format for docking. Because the exact binding pockets were not predefined, blind docking was performed by setting the grid box to cover the whole protein. Docking was carried out using AutoDock Vina 1.2.5, and repeated docking runs were performed for each ligand–target pair. The pose with both favorable binding energy and high reproducibility was selected for subsequent visualization and interaction analysis using PyMOL 3.1 and Discovery Studio.

### 4.13. Cellular Thermal Shift Assay (CETSA)

To assess the interaction between HJSS-derived small molecules and JNK, a cellular thermal shift assay was performed. HJSS granules was passed through a 10 kDa ultrafiltration membrane (FUF510, Beyotime) to obtain low-molecular-weight fractions. Treated gastric cancer cells were incubated with or without the HJSS ultrafiltration fraction for a defined period, harvested, and resuspended in PBS containing protease inhibitors. Cell suspensions were aliquoted into PCR tubes and heated at a series of temperatures (44–65 °C) for a fixed time, then rapidly cooled on ice. After lysis and centrifugation, the supernatants were collected and analyzed by Western blot to detect soluble JNK. Thermal stability shifts in the presence of HJSS fractions were interpreted as evidence consistent with altered JNK target engagement.

### 4.14. Statistical Analysis

Data were analyzed with GraphPad Prism 8.0, and each experiment was performed at least three times. Results are presented as mean ± SEM. One-way ANOVA was applied for normally distributed data with equal variance, while Welch’s ANOVA or non-parametric tests were used otherwise. Statistical significance was defined as *p* < 0.05.

## 5. Conclusions

In conclusion, HJSS attenuates inflammation-enhanced gastric cancer metastasis and EMT in both in vivo and in vitro models. Our results highlight a JNK-centered mechanism, in which HJSS suppresses JNK phosphorylation with a pronounced reduction in nuclear p-JNK accumulation under inflammatory stress. These findings support HJSS as a mechanistically grounded, multi-target herbal intervention for inflammation-associated gastric cancer progression.

## Data Availability

Data available on request due to restrictions. The data presented in this study are available on request from the corresponding author due to regulatory requirements for human genetic resource data in China, the dataset is currently under controlled access and will be available upon approval by the Human Genetic Resource Management Platform of the Ministry of Science and Technology of China.

## Supplementary Materials

The following supporting information can be downloaded at: https://www.mdpi.com/article/10.3390/ph19040636/s1, Figure S1: Validation of key bioactive compounds and JNK pathway involvement in HJSS-mediated regulation of EMT. Table S1: Identification of the main components in HJSS extract; Table S2: Detection of representative docking compounds in HJSS; Table S3: Annotation evidence for representative major constituents identified in HJSS by UPLC-QTOF-MS; Table S4: Information on antibodies; Table S5: Primers used for qRT–PCR analysis.

## Abbreviations

The following abbreviations are used in this manuscript:

Abbreviations	Full Name
GC	Gastric cancer
EMT	Epithelial-mesenchymal transition
JNK	c-Jun N-terminal kinase
MAPK	mitogen-activated protein kinase
5-FU	5-fluorouracil
LPS	Lipopolysaccharide
TCM	Traditional Chinese Medicine
HJSS	Huangjin Shuangshen Granules
HE	Hematoxylin-Eosin
CCK-8	Cell Proliferation Assay
IHC	Immunohistochemistry
IF	Immunofluorescence
WB	Western Blotting
qPCR	Quantitative Real-Time PCR
PPI	protein–protein interaction
STAT3	signal transducer and activator of transcription 3
TNF	tumor necrosis factor
IL-6	interleukin-6
DEGs	differentially expressed genes
GSEA	Gene set enrichment analysis
TME	tumor microenvironment
TLR4	Toll-like receptor 4
