# Supplementary material for: Inflammation-Driven JNK Activation Promotes EMT and Metastasis in Gastric Cancer and Is Attenuated by Huangjin Shuangshen Granules
Source: Pharmaceuticals (Basel). 2026 Apr 17;19(4):636. doi: 10.3390/ph19040636 (PMC13119064; doi:10.3390/ph19040636)
Supplement: Supplementary file 1 [file pharmaceuticals-19-00636-s001.zip › pharmaceuticals-4214216-supplementary.pdf]

Table S1. Identification of the main components in HJSS extract.

Rank	Metabolites	Formula	Ion mode	m/z	RT (min)	Score	Rel. abundance (%)	Source herb
1	Salvianolic acid Y	C36H30O16	NEG	717.14774	20.56	74.55	10.74086966	Salvia
2	Secologanic acid	C16H22O10	NEG	373.11442	12.124	73.05	10.38564657	Lonicera
3	Chlorogenic acid	C16H18O9	NEG	353.08808	11.494	68.75	8.092647846	Salvia, Astragalus, Glycyrrhiza, Angelica, Scrophularia, Lonicera
4	Neochlorogenic acid	C16H18O9	NEG	353.08815	9.248	74.69	6.313463219	Lonicera, Angelica
5	Syringaldehyde	C9H10O4	POS	165.05485	15.606	56.3	5.923278311	Salvia, Astragalus, Glycyrrhiza, Scrophularia
6	Cryptochlorogenic acid	C16H18O9	NEG	353.08813	11.948	73.78	5.922259736	Lonicera, Angelica, Scrophularia
7	Secologanin	C17H24O10	POS	411.12595	15.597	72.74	4.864567632	Lonicera
8	Guaiacol	C7H8O2	POS	107.04959	15.597	62.36	4.234150631	Salvia, Angelica, Glycyrrhiza, Scrophularia
9	Isochlorogenic acid C	C25H24O12	NEG	515.11916	19.951	75.67	3.997849143	Lonicera
10	Sweroside	C16H22O9	NEG	403.12413	13.579	73.55	3.602659289	Lonicera
11	Rosmarinic acid	C18H16O8	NEG	359.0777	19.966	76.85	2.763496458	Salvia
12	Turanose	C12H22O11	POS	365.10506	0.988	69.8	2.724607474	Salvia, Astragalus, Angelica, Glycyrrhiza, Scrophularia
13	N-Feruloyltyramine	C18H19NO4	POS	336.12259	20.719	57.33	2.233900912	Scrophularia
14	Trigonelline	C7H7NO2	POS	138.0551	0.797	75.96	2.201659277	Astragalus, Glycyrrhiza
15	Cryptotanshinone	C19H20O3	POS	319.13086	31.366	70.08	2.072624427	Salvia
16	Danshensu	C9H10O5	NEG	197.04518	7.116	72.11	1.797817865	Salvia
17	Calycosin	C16H12O5	POS	285.07554	21.132	73.31	1.532756663	Salvia, Astragalus, Angelica, Glycyrrhiza
18	Formononetin	C16H12O4	POS	269.08103	23.582	72.11	1.514466759	Salvia, Astragalus, Angelica, Glycyrrhiza
19	Ononin	C22H22O9	POS	431.13335	20.49	73.72	1.466297407	Astragalus, Glycyrrhiza, Angelica
20	Secoxyloganin	C17H24O11	POS	427.12082	15.025	74.32	1.426086692	Salvia, Lonicera, Scrophularia
21	Harpagoside	C24H30O11	NEG	539.17784	21.12	73.15	1.165457476	Astragalus, Angelica, Scrophularia
22	Harpagide	C15H24O10	NEG	363.13012	8.555	76.59	1.125970023	Scrophularia
23	Pipecolinic acid	C6H11NO2	POS	130.08676	1.209	66.59	0.875656264	Salvia, Angelica, Scrophularia
24	Levoglucosan	C6H10O5	POS	163.06007	1.057	69.4	0.574962057	Salvia, Astragalus, Scrophularia
25	Licoisoflavone A	C20H18O6	NEG	353.10336	28.78	71.85	0.573316367	Glycyrrhiza
26	Pseudocoptisine	C19H14NO4+	POS	320.09143	19.906	66	0.510566288	Salvia
27	Loganin	C17H26O10	POS	413.14097	13.844	68.26	0.484511248	Lonicera
28	Licorice-saponin H2	C42H62O16	NEG	821.39959	26.098	64.88	0.47376837	Glycyrrhiza
29	Dihydrotanshinone I	C18H14O3	POS	279.10159	29.615	73.3	0.470409625	Salvia
30	Isoquercitrin	C21H20O12	NEG	463.08927	17.639	71.68	0.463932673	Salvia

Table S2. Detection of representative docking compounds in HJSS.

Docking compound	Ion mode	RT (min)	m/z	Score	Relative abundance (%)	Notes
Kaempferol	NEG	22.129	285.04037	57.01	0.006575549	Direct match; used for docking with MAPK8 (JNK)
Quercetin	NEG	21.05	301.03574	55.1	0.01193385	Direct match; used for docking with IL1B
Luteolin	NEG	21.057	285.0411	68.84	0.158538161	Direct match; used for docking with AKT1
Cryptotanshinone	POS	31.366	319.13086	70.08	1.112601283	Direct match; used for docking with RELA
Isorhamnetin	NEG	22.44	315.05126	55.72	0.00267944918003897	Direct match; used for docking with PRKACA

Table S3. Annotation evidence for representative major constituents identified in HJSS by UPLC-QTOF-MS.

Compound	Formula	Ion mode	Adduct	Retention time (min)	Theoretical precursor m/z	Experimental precursor m/z	Mass error (ppm)	Major fragment ions (experimental)	Annotation level	Annotation basis
Salvianolic acid Y	C36H30O16	NEG	M-H	20.560	717.14698	717.14774	1.054	295.0607, 321.0404, 339.0508, 519.0917, 537.1027, 699.1312	level1	Annotated by LuMet-TCM standard database matching based on accurate mass, RT and characteristic MS/MS fragments
Secologanic acid	C16H22O10	NEG	M-H	12.124	373.11348	373.11442	2.513	123.0430, 137.0222, 165.0536, 193.0481, 211.0582, 329.1235	level1	Accurate mass + RT + MS/MS fragments matched to database reference
Chlorogenic acid	C16H18O9	NEG	M-H	11.494	353.08781	353.08808	0.772	93.0332, 135.0441, 173.0431, 179.0334, 191.0550	level1	Accurate mass + characteristic caffeoylquinic acid fragment ions + database match
Neochlorogenic acid	C16H18O9	NEG	M-H	9.248	353.08781	353.08815	0.960	93.0332, 111.0435, 135.0439, 173.0432, 191.0548	level1	Accurate mass + RT + characteristic fragment ions + database match
Cryptochlorogenic acid	C16H18O9	NEG	M-H	11.948	353.08781	353.08813	0.904	93.0331, 111.0435, 135.0438, 173.0431, 191.0548	level1	Accurate mass + RT + characteristic fragment ions + database match
Isochlorogenic acid C	C25H24O12	NEG	M-H	19.951	515.11911	515.11916	0.096	135.0439, 173.0431, 179.0334, 191.0547, 353.0877	level1	Accurate mass + dicaffeoylquinic acid fragment ions + database match
Rosmarinic acid	C18H16O8	NEG	M-H	19.966	359.07724	359.07770	1.276	133.0282, 135.0436, 161.0230, 179.0332, 197.0440	level1	Accurate mass + RT + characteristic phenolic acid fragments + database match
Cryptotanshinone	C19H20O3	POS	M+H	31.366	297.14852	297.14871	0.638	251.1426, 254.0737, 279.1378, 282.0670	level1	Accurate mass + tanshinone-type fragments + database match
Danshensu	C9H10O5	NEG	M-H	7.116	197.04555	197.04518	-1.878	123.0440, 135.0439, 153.0180, 179.0334	level1	Accurate mass + characteristic fragment ions + database match
Luteolin	C15H10O6	NEG	M-H	21.057	285.04046	285.04110	2.240	113.0234, 133.0284, 151.0029, 175.0387, 199.0393, 217.0497, 241.0500	level1	Accurate mass + flavone fragment ions + database match
Quercetin	C15H10O7	NEG	M-H	21.050	301.03537	301.03574	1.196	107.0129, 121.0284, 151.0027, 178.9976, 229.0493, 273.0404	level2	Putative annotation based on accurate mass and MS/MS fragment matching
Kaempferol	C15H10O6	NEG	M-H	22.129	285.04046	285.04037	-0.315	151.0029, 153.0188, 185.0236, 229.0493, 257.0442	level2	Putative annotation based on accurate mass and MS/MS fragment matching
Isorhamnetin	C16H12O7	NEG	M-H	22.440	315.05103	315.05126	0.731	151.0027, 165.0183, 193.0133, 300.0264	level2	Putative annotation based on accurate mass and MS/MS fragment matching

Table S4. Information on antibodies.

Antibody	Species	Source	Identifier	Dilution
E-cadherin	Rabbit	CST	3195S	1:1000 for WB; 1:500 for IHC
N-cadherin	Rabbit	Proteintech	22018	1:10000 for WB; 1:5000 for IHC
Vimentin	Rabbit	CST	5741S	1:500 for IHC
p38	Rabbit	CST	8690	1:1000 for WB
p-p38	Rabbit	CST	4511	1:1000 for WB
SAPK/JNK	Rabbit	CST	9252	1:1000 for WB
P-SAPK/JNK	Rabbit	CST	4668	1:1000 for WB
Phospho-JNK	Rabbit	Proteintech	80024	1:500 for IF
MKK4	Rabbit	Proteintech	17340-1-AP	1:1000 for WB
P-MKK4	Rabbit	Proteintech	29250-1-AP	1:1000 for WB
MKK7	Rabbit	Abclonal	A12950	1:1000 for WB
P-MKK7	Rabbit	Proteintech	80357-1-RR	1:2000 for WB
Lamin B1	Rabbit	Abclonal	A11495	1:3000 for WB
GAPDH	Mouse	Abclonal	AC033	1:100000 for WB
β-actin	Rabbit	Abclonal	AC026	1:150000 for WB

Table S5. Primers used for qRT–PCR analysis.

Gene Symbol	Primer Forward	Primer Reverse
E-cadherin	GCCTCCTGAAAAGAGAGTGGAAG	TGGCAGTGTCTCTCCAAATCCG
N-cadherin	CCTCCAGAGTTTACTGCCATGAC	GTAGGATCTCCGCCACTGATTC
Vimentin	CACGAAGAGGAAATCCGGAGC	CAGGGCGTCATTGTTCCG
Snail	CTTGTGTCTGCACGACCTGT	CTTCACATCCGAGTGGGTTT
Slug	TGTGACAAGGAATATGTGAGCC	TGAGCCCTCAGATTTGACCTG
Twist	GTCCGCAGTCTTACGAGGAG	CCAGCTTGAGGGTCTGAATC
ZEB1	CGCAGTCTGGGTGTAATCGTAA	GACTGCCTGGTGATGCTGAAA
18s	GAGGATGAGGTGGAACGTGT	AGAAGTGACGCAGCCCTCTA

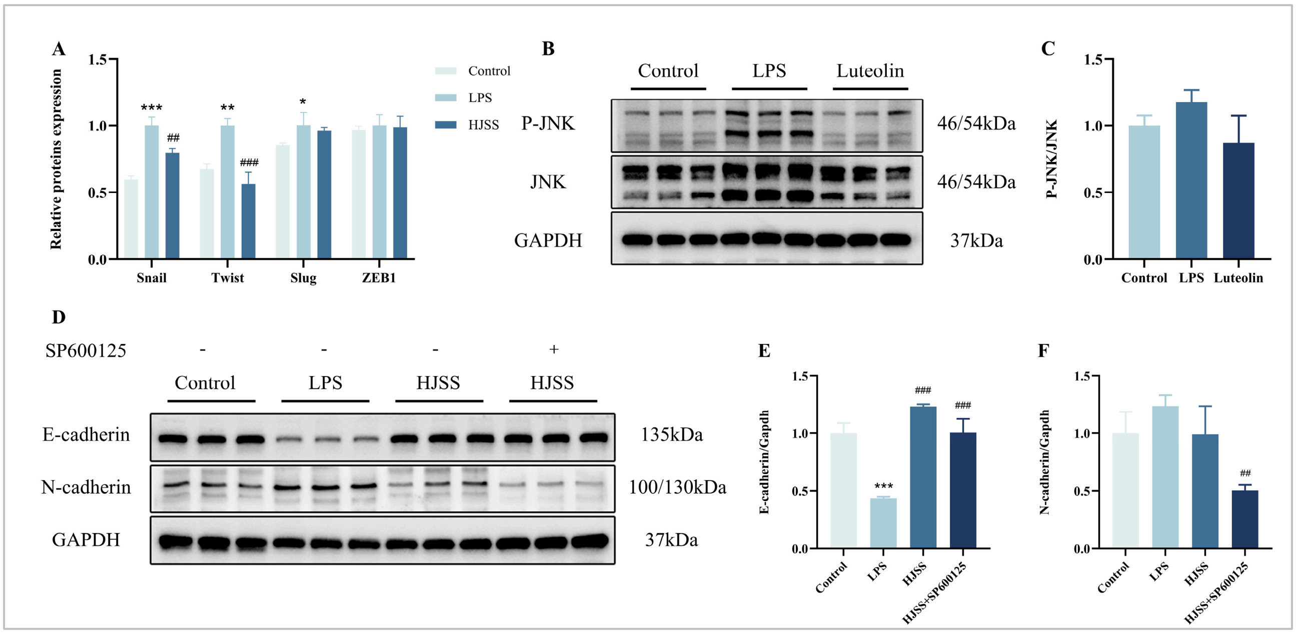

Figure S1. Validation of key bioactive compounds and JNK pathway involvement in HJSS-mediated regulation of EMT.

(A) RT-qPCR analysis of EMT-related transcription factors, including Snail, Twist, Slug, and ZEB1, in MKN-45 cells under the indicated treatments. (B) Representative Western blot analysis of phosphorylated JNK (p-JNK) and total JNK in MKN-45 cells treated with luteolin under LPS-stimulated conditions. (C) Densitometric quantification of the p-JNK/JNK ratio. (D) Representative Western blot analysis of E-cadherin and N-cadherin in MKN-45 cells treated with SP600125 under the indicated conditions. GAPDH was used as the loading control. (E-F) Densitometric quantification of E-cadherin/GAPDH and N-cadherin/GAPDH. Data are presented as mean ± SEM, $n = 3$. *$P < 0.05$, **$P < 0.01$, ***$P < 0.001$, vs. Control group, $^{\#\#}P < 0.01$, $^{\#\#\#}P < 0.001$, vs. LPS group.
